# Clinical effect of full endoscopic lumbar annulus fibrosus suture

**DOI:** 10.1186/s13018-024-04725-9

**Published:** 2024-04-24

**Authors:** Yin-xiao Peng, Yue Zhang, Yun Yang, Fei Wang, Bin Yu

**Affiliations:** 1 Department of Orthopaedics, The Third People’s Hospital of Chengdu, Sichuan, PR China; 2 Department of Neurosurgery, Pujiang People’s Hospital, Sichuan, PR China

**Keywords:** Lumbar disc herniation, Endoscopic discectomy, Annulus fibrous repair, Minimally invasive surgery

## Abstract

**Purpose:**

The aim of this study was to investigate the clinical efficacy of full endoscopic lumbar annulus fibrosus suture in the treatment of single-segment lumbar disc herniation (LDH).

**Methods:**

The clinical data of patients with single-segment LDH who underwent full endoscopic lumbar discectomy from January 2017 to January 2019 in our hospital were retrospectively analysed. Patients with full endoscopic lumbar discectomy combined with annulus fibrosus suture were divided into group A, and those with simple full endoscopic lumbar discectomy were divided into group B. The general information, surgery-related data, visual analog scale (VAS), Oswestry disability index (ODI), modified MacNab score at the last follow-up, reoperation rate and recurrence were compared between the two groups.

**Results:**

All patients were followed up for 12 to 24 months, and the surgical time was 133.6 ± 9.6 min in group A and 129.0 ± 11.7 min in group B. The difference was not statistically significant (*p* > 0.05). The blood loss of group A was higher than that of group B, and the difference was statistically significant when comparing the groups (*p* < 0.05). The postoperative symptoms of patients in both groups were significantly relieved, and the VAS score of low back pain and ODI index were significantly lower than the preoperative ones at all postoperative time points (1 month after surgery, 3 months after surgery, and at the last follow-up) (*p* < 0.05), but there was no significant difference between the groups (*p* > 0.05). The excellent rate of MacNab at the last follow-up in the two groups were 93.55% and 87.80%, respectively, with no statistically significant difference (*p* > 0.05). At the last follow-up, the recurrence rate of group A was significantly lower than that of group B, and the difference was statistically significant (*p* < 0.05), while the difference between the reoperation rate of the two groups was not statistically significant (*p* > 0.05).

**Conclusions:**

Full endoscopic lumbar discectomy combined with annulus fibrosus repair reduces the postoperative recurrence rate and achieves satisfactory clinical outcomes.

## Introduction

Lumbar disc herniation (LDH) is a condition in which the nucleus pulposus of the lumbar intervertebral disc protrudes or prolapses from the annulus fibrosus at the rupture of the fibrous annulus under the action of an external force, resulting in inflammatory irritation and nerve root compression, which in turn causes lumbar and leg pains and radiating pain in the sciatic nerve [1]. Nucleus pulposus removal surgery has become an effective treatment for LDH, but these surgical techniques are basically “annulus fibrosus destructive” surgeries [2, 3], with a certain recurrence rate and reoperation rate [4]. How to reduce the postoperative recurrence rate has been the focus of many scholars.

The reason for recurrence after nucleus pulposus removal is the destruction of the integrity of the annulus fibrosus, which makes the crater-like annular rupture expand out to mechanically irritate the nerve root in the short term after surgery. If the annular rupture heals poorly, the residual nucleus pulposus tissue in the disc protrudes into the vertebral canal at the original annular rupture again and compresses the nerve root, leading to the recurrence of herniated discs [5]. There are also studies that show that the long-term lumbar back pain after nucleus pulposus removal is related to the long-term release of inflammatory mediators by residual nucleus pulposus from the annular rupture to irritate the nerves of the back in the long term [6]. Bron et al. [6] reported a high recurrence rate and persistent postoperative low back pain after lumbar disc removal, and concluded that the fibrous rupture should be effectively treated after lumbar disc removal, such as tissue engineering reconstruction and fibrous annulus repair. Currently, bioremediation of the annulus fibrosus is still in the laboratory stage, including collagen modification, cell therapy, gene therapy and tissue engineering reconstruction [7–9]. Surgical repair techniques for lumbar fibrous annulus have been carried out in the clinic, including a variety of fibrous annulus suturing techniques and sealing techniques, and have achieved good results [10].

With the development of minimally invasive spinal technology, endoscopic lumbar discectomy has gradually become the main treatment procedure for LDH. In recent years, full endoscopic lumbar discectomy combined with annulus fibrosus suture technique has been gradually carried out in our unit for the treatment of LDH, and certain efficacy has been achieved. Therefore, the aim of this study was to investigate the efficacy of this surgical technique in the treatment of single-segment LDH.

## Materials and methods

### Subjects

Patients with single-segment LDH who underwent full endoscopic lumbar discectomy in our hospital from January 2017 to January 2019 were selected. Inclusion criteria were as follow: (i) single-segment disc herniation that had been ineffective with regular conservative treatment for 3 months or more; (ii) typical radiating pain in the lower limbs with or without lumbar pain; (iii) full endoscopic lumbar discectomy. Exclusion criteria included: (i) intervertebral disc prolapse with posterior rim dissection and disc calcification; (ii) lumbar slippage and instability; (iii) bony lumbar stenosis; (iv) severe degeneration of the intervertebral discs, significant narrowing of the intervertebral space, and end-plate inflammation of adjacent vertebral bodies; (v) previous spinal surgeries, combined with spinal infections, tumours, and fractures; and (vi) significant fibrous annulus defect after the intervertebral disc removal surgery. Patients who underwent simple full endoscopic lumbar discectomy were divided into group B, and those who underwent full endoscopic lumbar discectomy and fibrous annulus suture were divided into group A. The study was approved by the Ethics Committee of the hospital, and all patients signed the informed consent before surgery.

### Surgical process

X-rays, CT and MRI were routine preoperative investigations. The optimal approach was based on the preoperative imaging of the patients. For patients with central or paracentral LDH, the interlaminar approach was adopted; for patients with extreme lateral, posterolateral, or foraminal LDH, the transforaminal approach was adopted; and for L5/S1 segment, if the iliac bone was too high, the interlaminar approach was chosen. All patients were given local infiltration anaesthesia, and intraoperative sedative and analgesic drugs were given to assist the surgical treatment.

Group A: The patient was placed in the prone position and the responsible segment was localised by fluoroscopy. According to the pre-determined surgical approach, puncture was performed under fluoroscopic guidance. After confirming the good position, the guide wire was placed. The guide rods were placed step by step along the guide wire to expand the working channel, and then the working trocar and endoscope were placed. Foramen formation was performed through the transforaminal approach as needed according to the specific situation. The ligamentum flavum was removed to reveal the nerve root and the protruding disc. The annulus fibrosus was incised vertically in the direction of the interspace to reveal the disc, and the free and loose nucleus pulposus was removed from the disc. After opening the annulus fibrosus suture, a puncture needle was placed 2 mm from the edge of the annulus fibrosus breach. The puncture needle was inserted through the fibrous annulus and a first anchor was placed. The puncture needle was withdrawn after the puncture was in place. In the same way, another fixed anchor was placed on the opposite side of the annulus fibrosus breach. The first knot was tied externally and the knot was pushed into the disc with a knot pusher. The suture was then tightened to close the annulus fibrosus breach, maintaining a constant tension on the suture. A second knot was tied in the same way, and the free end of the suture was cut. Endoscopically, the fibrous ring breach was seen to be well sutured and tightly closed.

Group B: endoscopic discectomy was as above, but without fibrous annulus repair.

On the first postoperative day, straight leg raising exercise for lower limbs was started to prevent nerve root adhesion, and the patient got out of bed with a waist girdle. Functional exercises for lumbar and dorsal muscles were carried out 2∼3 weeks after surgery. Patients were required to wear a waist girdle for 1 month and were prohibited from stooping, strenuous activities and heavy labour for 3 months after surgery.

### Evaluation index

Surgical time, intraoperative blood loss and length of stay (LOS) were compared between the two groups. The patients’ visual analog scale (VAS) for low back pain and Oswestry disability index (ODI) were assessed preoperatively and at 1 month, 3 months and the final follow-up. At the final follow-up, the postoperative outcome was assessed with reference to the modified MacNab score. Postoperative complications, LDH recurrence and reoperation were also recorded.

### Statistical analysis

All data were analyzed using SPSS 25.0 statistical software (SPSS Chicago, IL, USA). Categorical variables were grouped and expressed as numerical values, and continuous data were expressed as mean ± standard deviation. Independent t-tests were used for between-group comparisons and paired t-tests for within-group comparisons. The counting data was done by χ2 test, and *p* < 0.05 was considered to indicate statistical significance.

## Results

### Patient characteristics

A total of 72 patients met the inclusion criteria. Among them, 31 patients were in group A and 41 patients were in group B. There was no statistically significant difference in the preoperative indicators between the two groups, and they were comparable (Table 1). All patients underwent the surgery successfully, and there was no significant intraoperative haemorrhage, nerve root injury, dural sac tear. There were no postoperative complications such as cerebrospinal fluid leakage, aggravation of neurological symptoms, and infection at the surgical site.

**Table 1:** Patients’ demographics

Variable	Group A	Group B	*p*
Age, years	35.7 ± 7.2	36.0 ± 12.2	0.907
Gender, n			0.088
Male	15	28	
Female	16	13	
BMI, kg/m^2^	22.5 ± 1.9	22.9 ± 1.8	0.284
Course of disease, months	13.1 ± 5.5	14.7 ± 5.6	0.227
Responsible levels			0.759
L4/5	11	16	
L5/S1	20	25	
Total	31	41	

BMI: Body mass index

### Clinical data

The average surgical time of group A was 133.6 ± 9.6 min, and the average surgical time of group B was 129.0 ± 11.7 min, the difference was not statistically significant (*p* > 0.05). The blood loss of group A was higher than that of group B, and the difference was statistically significant when comparing the groups (*p* < 0.05). The difference in the LOS of the two groups was not statistically significant (*p* > 0.05) (Table 2).

**Table 2:** Comparison of clinical data between the two groups

Variable	Group A	Group B	*p*
Surgical time, minutes	133.6 ± 9.6	129.0 ± 11.7	0.079
Blood loss, ml	44.7 ± 7.4	38.9 ± 6.5	0.001
LOS	5.2 ± 1.3	5.2 ± 1.7	0.986

LOS: Length of stay

### Comparison of treatment effect

All patients had immediate postoperative relief of lower limb radiating pain, and the VAS score of low back pain and ODI index at different time points after the operation of the two groups improved significantly compared with the preoperative period, and the difference was statistically significant (*p* < 0.05). The difference was not statistically significant when comparing the same period of time before and after the operation (*p* > 0.05) (Table 3). At the last follow-up, the postoperative efficacy was evaluated with reference to the modified MacNab score, and the excellent rate was 93.55% in group A and 87.80% in group B. There was no statistical significance in the comparison of the excellent rate of the two groups (*p* > 0.05) (Table 4).

**Table 3:** Comparison of VAS and ODI between the two groups

Variable	Follow-up time point	Group A	Group B	t	*p*
VAS	Preoperative	6.3 ± 0.9	6.4 ± 0.9	−0.736	0.464
	One month after surgery	3.5 ± 0.9	3.4 ± 0.8	0.681	0.498
	Three months after surgery	2.6 ± 0.7	2.8 ± 0.6	−1.439	0154
	Last time	1.2 ± 0.7	1.2 ± 0.7	-0.111	0.912
ODI	Preoperative	60.1 ± 5.7	61.0 ± 6.8	-0.571	0.570
	One month after surgery	38.1 ± 3.1	38.2 ± 2.9	-0.206	0.838
	Three months after surgery	28.9 ± 3.2	30.0 ± 2.3	-1.788	0.078
	Last time	19.2 ± 1.7	18.7 ± 2.4	0.888	0.377

VAS: visual analog scale; ODI: Oswestry disability index

**Table 4:** Comparison of the results of the modified MacNab score at the last follow-up between the two groups

Group	n	Excellent	Good	Fair	Poor
A	31	26	3	2	0
B	41	33	3	4	1
χ^2^	1.130				
*p*	0.770				

### Comparison of recurrence rate and reoperation rate

Patients in both groups were followed up for 12 to 24 months. According to the definition of recurrent lumbar disc herniation [11], from 6 months after surgery to the last follow-up, there was no case of recurrence in group A, while there were 5 cases of recurrence in group B, with a recurrence rate of 12.2%, of which 3 patients were reopened after ineffective conservative treatment, with a reoperation rate of 7.3%. Comparison of postoperative recurrence and reoperation between the two groups showed that the recurrence rate of group A was significantly lower than that of group B, and the difference was statistically significant (*p* < 0.05). In contrast, the difference between the reoperation rate of the two groups was not statistically significant *(p* > 0.05) (Table 5).

**Table 5:** Postoperative recurrence and reoperation

Group	n	Recurrence	Reoperation
A	31	0	0
B	41	5	3
χ^2^		4.063	2.367
*p*		0.044	0.124

## Discussion

Simple lumbar discectomy is still the main surgical method for the treatment of LDH, and the clinical results are relatively satisfactory. However, the postoperative recurrence rate is still high. It has been reported that there is a 5-19% recurrence rate after LDH [4]. Carragee et al. [12] pointed out that the recurrence rate after nucleus pulposus removal is related to the integrity of the annulus fibrosus. Poor closure of the fibrous annulus incision or rupture after nucleus pulposus removal alone may lead to re-projection of residual nucleus pulposus tissue through the original fibrous annulus rupture. In addition, some studies have shown that after nucleus pulposus removal, because the ruptured fibrous ring is not closed, the residual nucleus pulposus releases inflammatory factors to stimulate the nerves of the lumbar back in the long term, resulting in long-term low back pain [6]. In view of this, in order to prevent the residual nucleus pulposus from protruding from the broken annulus after lumbar nucleus pulposus removal, some surgeons have tried to remove as much residual nucleus pulposus as possible during the operation, so as to reduce the re-protrusion of the residual nucleus pulposus after the operation. However, the excessive removal of nucleus pulposus causes further damage to the intervertebral disc structure, which accelerates disc degeneration and leads to disc narrowing and intervertebral space collapse, resulting in postoperative lumbar vertebral instability and long-term lumbar and back pain [13].

In order to reduce the recurrence of LDH, some scholars have tried to repair the rupture of the annulus fibrosus with suture. Bailey et al. [14] used Xclose Tissue Repair System to close the annulus fibrosus and followed up more than 750 patients, which showed that the recurrence rate of the suture group could be effectively reduced in 6 months after the operation, and the reoperation rate in the 2-year period after the operation was significantly reduced by 45%. Animal experiments have also shown that suturing the ruptured annulus fibrosus can promote the healing of the annulus fibrosus, effectively prevent the nucleus pulposus from protruding again, and also increase the anti-mechanical properties of the intervertebral discs, effectively avoiding disc degeneration due to excessive removal of the nucleus pulposus, and maintaining the biomechanical strength of the intervertebral discs [15].

In the group of 31 patients with LDH who underwent full endoscopic lumbar discectomy combined with annulus fibrosus suture, there was no recurrence of disc herniation during the follow-up period. Compared with discectomy alone, the recurrence rate was significantly lower (*p* < 0.05). This confirmed the effect of fibrous annulus suture repair in reducing recurrence after lumbar nucleus pulposus removal. However, there was no statistically significant difference between the two groups in terms of reoperation rate, which may be related to the small sample size. Blood loss increased in group A compared to group B, and the difference was statistically significant (*p <* 0.05). This indicates that intraoperative annulus fibrosus suture increases the invasiveness of the operation. However, there was no significant difference in surgical time and LOS between the two groups, suggesting that annulus fibrosus suture does not increase the complexity of the operation or delay hospital discharge. Compared with the preoperative period, the postoperative VAS scores and ODI of the patients in the two groups were significantly improved (*p* < 0.05), and there was no significant difference in the above indexes between the two groups at each time point (*p* > 0.05). The difference in the MacNab excellence rate of the patients in the two groups at the last postoperative follow-up was statistically insignificant (*p* > 0.05), which indicated that intraoperative annulus fibrosus suture did not have any significant effect on the patients’ outcomes.

For those who initially perform endoscopic annulus fibrosus repair, there are certain challenges. The problem of multiple punctures during the operation results in burrs on the edges of the annulus fibrosus and a decrease in the strength of the suture, which fails to rebuild the mechanical integrity of the annulus fibrosus, resulting in a poorly closed annulus fibrosus rupture or suture dislodgement, which affects the effectiveness of the operation. In order to improve the success rate of endoscopic annulus fibrosus repair, the following points should be noted: (i) If the annulus fibrosus rupture is found to be large or the first suture is ineffective, the second suture can be crossed again or parallel to ensure that the rupture of the annulus fibrosus is well closed; (ii) It is appropriate to puncture the needle from the edge of the rupture of the annulus fibrosus by more than 2 mm during the operation, so as to keep the good biomechanical properties of the annulus fibrosus, and to avoid excessive tension in the suture thread. (iii) If the disc herniation is of the inclusive type, a linear incision of about 5 mm should be made; otherwise, a large incision will cause excessive damage to the annulus fibrosus and affect the closure, while a small incision will make it difficult to remove the nucleus pulposus, increase the difficulty of intraoperative operation, and increase the probability of injury to the annulus fibrosus; (iv) Intraoperative operation should be performed softly and gently to reduce the damage to the annulus fibrosus and improve the success rate of the closure. (v) Suture should be abandoned in cases where the annulus fibrosus is too large to be sutured.

This study had some limitations. Firstly, this study was a single-centre retrospective study; Secondly, the small sample size and short follow-up period made it prone to selection bias and recall bias. Future prospective studies with large samples and multiple centres will be conducted to further validate our findings.

## Conclusion

Full endoscopic lumbar discectomy combined with annulus fibrosus repair for single-segment LDH can reconstruct the integrity of the annulus fibrosus, reduce the postoperative recurrence rate, and achieve satisfactory clinical outcomes, which is worthy of clinical application.

## Data Availability

No datasets were generated or analysed during the current study.

## Abbreviations

VAS: visual analog scale ODI: Oswestry disability index
LDH: Lumbar disc herniation LOS: Length of stay
